# Optimizing HIV-1 Genotypic Resistance Testing for Low- and Middle-Income Countries: High-Impact HIV-1 Mutations Across WHO-Defined Scenarios

**DOI:** 10.3390/v18060588

**Published:** 2026-05-22

**Authors:** Robert W. Shafer, Kaiming Tao, Tom Loosli, Ana Abecasis, Daniele Armenia, George Bwire, Ricardo Sobhie Diaz, Joseph Fokam, Amalia Giron, Seth Inzaule, Rami Kantor, Cissy Kityo, Roger D. Kouyos, Swarali Kurle, Anne-Genevieve Marcelin, Roger Paredes, Martine Peeters, Victor F. Pimentel, Jonathan M. Schapiro, Kim Steegen, Marco Vitoria, Annemarie M. Wensing, Neil Parkin, Michael R. Jordan

**Affiliations:** 1 Division of Infectious Diseases, Department of Medicine, Stanford University Stanford, Stanford, CA 94305, USA; 2 Department of Infectious Diseases and Hospital Epidemiology, University Hospital Zurich, 8091 Zurich, Switzerland; 3 Global Health and Tropical Medicine, Associate Laboratory in Translation and Innovation Towards Global Health, LA-REAL, Instituto de Higiene e Medicina Tropical, IHMT, Universidade NOVA de Lisboa, UNL, 1349-008 Lisbon, Portugal; 4 Department Faculty, Saint Camillus International University of Health Sciences, 00131 Rome, Italy; 5 Department of Pharmaceutical Microbiology, Muhimbili University of Health and Allied Sciences, Dar es Salaam P.O. Box 65001, Tanzania; 6 Infectious Diseases Division, Department of Medicine, Federal University of São Paulo, São Paulo 04023-062, Brazil; 7 Chantal BIYA International Reference Centre for Research on HIV/AIDS Prevention and Management (CIRCB), Yaounde P.O. Box 3077, Cameroon; josephfokam@gmail.com; 8 Independent Consultant, Guatemala City 01010, Guatemala; 9 Department of Global Health, Amsterdam Institute for Global Health and Development, University of Amsterdam, 1105 AZ Amsterdam, The Netherlands; 10 Division of Infectious Diseases, Department of Medicine, Brown University, Providence, RI 02903, USA; rkantor@brown.edu; 11 Joint Clinical Research Centre, Kampala P.O. Box 10005, Uganda; 12 HIV Drug Resistance Laboratory, Indian Council of Medical Research—National Institute of Translational Virology and AIDS Research, Pune 411026, India; 13 Sorbonne Université, INSERM, Institut Pierre Louis d’Epidémiologie et de Santé Publique, AP-HP, Hôpitaux Universitaires Pitié-Salpêtrière-Charles Foix, Laboratoire de Virologie, 75013 Paris, France; 14 Department of Infectious Diseases and irsiCaixa, Hospital Universitari Germans Trias i Pujol, 08916 Badalona, Spain; 15 Recherches Translationnelles sur le VIH et Maladies Infectieuses, Université de Montpellier/INSERM U1175, Institut de Recherche pour le Développement, 34394 Montpellier, France; 16 National Hemophilia Center, Sheba Medical Center, Ramat Gan 5265601, Israel; 17 Department of Molecular Medicine and Haematology, School of Pathology, Faculty of Health Sciences, University of the Witwatersrand, Johannesburg 2193, South Africa; 18 Department for HIV, Tuberculosis, Hepatitis, and Sexually Transmitted Infections, World Health Organization, 1211 Geneva, Switzerland; 19 Department of Global Health and Bio-Ethics, Julius Center for Health Sciences and Primary Care, University Medical Center, 3584 CX Utrecht, The Netherlands; 20 Ezintsha, Wits RHI, University of the Witwatersrand, Johannesburg 2193, South Africa; 21 Independent Researcher, Sebastopol, CA 95473, USA; nparkin34@gmail.com; 22 Division of Geographic Medicine and Infectious Diseases, Tufts Medical Center, Boston, MA 02111, USA

**Keywords:** Antiretroviral drugs, HIV drug resistance, HIV mutations

## Abstract

Introduction: Drug resistance testing may improve the management of people living with HIV (PLWH) in several scenarios in low- and middle-income countries (LMICs). To guide assay development, the WHO published a target product profile (TPP) outlining two priority use cases (scenarios) for genotypic resistance testing: (1) PLWH with confirmed virological failure (VF) on an integrase strand transfer inhibitor (INSTI)-based regimen, such as tenofovir (TFV) disoproxil fumarate, lamivudine (3TC), and dolutegravir (DTG) and (2) heavily treated PLWH, including infants and young children, with confirmed VF after receiving multiple regimens including a boosted protease inhibitor (PI). An additional potential scenario includes PLWH testing positive for HIV-1 while on pre-exposure prophylaxis (PrEP). Methods: To identify drug-resistance mutations (DRMs) most likely to influence clinical management of PLWH in each WHO TPP scenarios and to inform development of assays that detect individual DRMs and the interpretation of sequence-based assays, we reviewed prevalence and in vitro susceptibility data on HIV-1 DRMs in the Stanford HIV Drug Resistance Database associated with the nucleoside RT inhibitor (NRTI), nonnucleoside RT inhibitor (NNRTI), PI, and INSTI classes and the capsid inhibitor lenacapavir. Results: In the first scenario, the most informative NRTI DRMs were K65R and M184V/I; and the most informative INSTI DRMs were G118R, N155H, Q148H/K/R, and R263K. In the second scenario, a broader spectrum of DRMs is likely to be clinically relevant, including additional NRTI DRMs, the PI DRMs associated with reduced susceptibility to darunavir, and the NNRTI DRMs associated with reduced susceptibility to etravirine and doravirine. In PLWH testing positive for HIV-1 despite PrEP, the most informative NRTI and INSTI DRMs overlap with those in the first scenario, together with the capsid DRMs reported in persons experiencing VF while receiving lenacapavir. Conclusions: As global ART programs increasingly rely on INSTI-based regimens, and as the number of heavily treated individuals and difficult-to-treat pediatric cases grows, many LMICs have begun introducing HIV drug resistance testing for patient management. Although sequence-based assays provide the most comprehensive information for managing individual PLWH, assays that detect individual DRMs are also likely to be highly useful in the three WHO TPP scenarios.

## 1. Introduction

HIV-1 genotypic drug resistance testing is routinely used in high-income countries to guide antiretroviral therapy (ART) for people living with HIV (PLWH). In low- and middle-income countries (LMICs), genotypic resistance testing has primarily been performed for surveillance to generate population-level estimates of pre-treatment and acquired HIV drug resistance (HIVDR) [1,2]. Recently, there has been growing interest in the feasibility and potential utility of drug resistance testing for selected clinical scenarios in LMIC settings [3].

In LMICs, sequencing capacity has expanded following the scale-up of HIVDR surveillance programs with Sanger-based sequencing protocols demonstrating reliable performance across reference laboratories [1]. Additional technologies, including next-generation sequencing [4] and point mutation assays targeting specific codons, have also been developed [5,6]. To guide the development of low-cost assays by diagnostic manufacturers, the World Health Organization (WHO) published a target product profile (TPP) for HIVDR testing in 2023 [3]. To support this effort, this review synthesizes HIVDR mutation data to inform development of targeted assays designed to detect individual DRMs and help interpret sequence-based assays.

The WHO TPP outlines two priority use cases (scenarios) for genotypic resistance testing. The first involves PLWH with confirmed virological failure (VF) on an integrase strand transfer inhibitor (INSTI)-based regimen most often dolutegravir (DTG) in combination with tenofovir (TFV) disoproxil fumarate and lamivudine (3TC), as the fixed dose combination TLD [2]. Although DTG resistance is fairly uncommon among PLWH with VF on first-line TLD [2,7,8], prevalence estimates ≥20% have been reported among those receiving second-line DTG-containing regimens [2,9,10,11]. In this scenario, resistance testing helps distinguish VF caused by suboptimal adherence from VF caused by HIVDR. The detection of clinically relevant DTG resistance mutations should prompt a regimen change in accordance with national treatment guidelines, whereas those without DTG resistance may benefit from enhanced adherence counseling [12].

The second scenario involves heavily treatment experienced PLWH with VF following treatment with antiretrovirals (ARVs) from multiple drug classes including protease inhibitors (PIs), for whom resistance testing can help design salvage ART regimens [13,14,15]. This scenario is also relevant for choosing therapy for infants and young children, who often exhibit higher HIVDR rates due to perinatal transmission of resistant virus and fewer treatment options [2].

An additional, lower-priority use case proposed by the WHO TPP, defined in this work as a third scenario, involves persons who test positive for HIV while receiving pre-exposure prophylaxis (PrEP), placing them at risk of selecting resistance to the ARVs used for PrEP with potential cross-resistance to the ARVs used for treatment [16].

This review focuses on five ARV classes targeting four HIV proteins: reverse transcriptase (RT) for nucleoside RT inhibitors (NRTIs) and nonnucleoside RT inhibitors (NNRTIs), protease for protease inhibitors (PIs), integrase for INSTIs, and capsid for lenacapavir. Entry inhibitors are not discussed, as they remain largely unavailable in LMICs and their mutations are often difficult to define. We first review the DRMs associated with each of the five ARV classes and then synthesize these data in the context of the three WHO TPP clinical scenarios.

## 2. Methods

### 2.1. ARVs

This review focuses on ARVs likely to have been used previously by PLWH in the different WHO TPP scenarios, as well as ARVs likely to be used now or potentially in the future in a regimen following genotypic resistance testing. The drugs considered include: (1) the three commonly used dual-NRTI combinations—abacavir (ABC) plus 3TC, TFV plus 3TC or FTC (XTC), and zidovudine (AZT) plus 3TC; (2) the second-generation INSTIs cabotegravir (CAB), bictegravir (BIC), and DTG; (3) the NNRTIs doravirine (DOR), efavirenz (EFV), etravirine (ETR), and rilpivirine (RPV); (4) the PIs atazanavir (ATV), darunavir (DRV), and lopinavir (LPV). ATV, DRV, and LPV refer to the corresponding PIs, regardless of whether they were administered with ritonavir or cobicistat boosting; and (5) the capsid inhibitor lenacapavir (LEN). While ETR, RPV, DOR, and BIC are not included in the latest WHO treatment guidelines [17], they are being actively studied in multiple LMICs [13,14,15,18,19,20,21,22,23,24,25]. Islatravir, an NRTI approved by the U.S. Food and Drug Administration in April 2026 as part of a fixed-dose combination with doravirine for switch therapy in virologically suppressed PLWH, was not reviewed due to insufficient available data.

### 2.2. DRMs

DRMs were initially defined as those with a mutation penalty score in the Stanford HIV Drug Resistance Database (HIVDB) interpretation program (version 10.0, updated December 2025) for ARVs likely to be used in one of the WHO TPP scenarios. The initial list for each drug class was then refined using two additional criteria: included DRMs were required to be nonpolymorphic defined as occurring in fewer than one percent of PLWH in the absence of selective drug pressure and to occur in at least one percent of PLWH in one of the WHO TPP scenarios. Several exceptions to these criteria were made based on clinical considerations outlined in the corresponding sections for each drug class.

We did not review published data on mutations outside the molecular targets of currently approved ARVs [26]. One notable area of ongoing research involves mutations in the HIV-1 nucleocapsid protein that emerge under INSTI selective pressure and contribute to reduced INSTI susceptibility by accelerating the kinetics of viral DNA integration, thereby shortening the time window during which INSTIs can block integration [27].

### 2.3. Data Sources

To estimate the prevalence of DRMs in different treated populations, we used data from HIVDB. As of 1 February 2026, HIVDB had genotypic data from 2304 references of which 95% were published studies and 5% were conference presentations or GenBank submissions without an associated publication. Genotypic data were available from approximately 321,000 PLWH of whom 98.8% had nucleotide sequences and 1.2% had complete or partial amino acid lists. Treatment data were obtained from published studies, conference proceedings, and GenBank submissions. The dataset represents geographically diverse populations, including a high proportion from LMICs.

Sequences were obtained from PLWH who were either ART-naïve or who had developed VF following treatment with one or more ARVs. Approximately 95% of sequences from individuals with VF were obtained from plasma, 4% from peripheral blood mononuclear cells, and 1% from dried blood spots. Among ART-naïve individuals, it was not possible to exclude those who had acquired drug-resistant viruses through transmission (i.e., transmitted drug resistance).

To estimate the impact of specific DRMs on in vitro ARV susceptibility, we analyzed HIVDB data from approximately 12,600 RT, protease, integrase, and capsid isolates. Publications addressing the impact of DRMs on the virological response to ART were identified by the authors based on their expert knowledge of the field. The phenotypic data used for the paper, as well as data from publications were also obtained through 1 February 2026.

This study did not involve human participants, human data, or human tissue. The research was based entirely on data from previously published studies. As such, ethical approval and informed consent were not required.

### 2.4. DRM Prevalence Estimates

For each DRM, we estimated its prevalence among PLWH who had received only a single ARV from the relevant drug class. For example, to identify DRMs associated with the NNRTI RPV, we analyzed genotypic data from PLWH treated with RPV but no other NNRTIs. We made two exceptions to this approach. First, because NRTIs are usually used in combination, NRTI DRM prevalences were calculated for individuals receiving each of three common dual NRTI combinations. Second, because CAB is primarily used in PLWH with virological suppression on prior oral therapy, we also included genotypic data from persons previously treated with another INSTI, provided they had not experienced VF on that INSTI.

For the INSTIs BIC and CAB and the NNRTI DOR, most genotypic data consisted of author-reported DRM lists rather than nucleotide sequences. To accommodate variability across studies—some studies including all individuals with viremia, others only those with a DRM—each DRM’s prevalence was reported among viruses with at least one DRM. Prevalences were calculated across all HIV-1 group M isolates combined, without stratification by subtype or recombinant form.

### 2.5. Phenotypic Susceptibility Data

HIVDB includes phenotypic susceptibility results generated using several assays, the most common being the PhenoSense assay (Monogram Biosciences; South San Francisco, CA, USA) [28], which has demonstrated high reproducibility [29,30]. Whenever sufficient PhenoSense data were available for a given ARV, analyses were restricted to those results. Exceptions were made for the INSTI CAB and the NNRTI DOR, for which most available phenotypic data were generated using other assays.

We used least squares regression to estimate the effect (coefficient) of individual DRMs on the log_10_-fold reduction in virus susceptibility. For each ARV, the regression models included the set of DRMs defined in the Methods provided that each DRM occurred in at least three virus sequences with susceptibility results. Ten repetitions of 5-fold cross-validation were performed to estimate the variance among the fitted coefficients. Because mixtures at drug-resistance positions may confound genotype-phenotype correlations (i.e., the effect of a DRM may be underestimated if the wild-type variant was also present), sequences containing mixtures at a DRM position were excluded from analysis.

For the NRTI, INSTI, and NNRTI classes, we constructed tables summarizing the median reductions in susceptibility of viruses containing common or illustrative DRM patterns. However, because the PI DRMs occurred in complex overlapping combinations, often involving multiple mutations each exerting modest effects on susceptibility, we were unable to construct an informative summary table for this drug class.

## 3. Results and Discussion

### 3.1. NRTI DRMs

We included nonpolymorphic DRMs that had a HIVDB mutation penalty score for TFV, XTC, or ABC and a prevalence ≥ 1% in persons receiving either TFV/XTC, ABC/3TC, or AZT/3TC. Exceptions were made for T69 insertions and Q151M, which although extremely uncommon were included because they are associated with high-level resistance to multiple NRTIs. This yielded a set of 25 DRMs at 13 positions: M41L, A62V, K65R/N, D67G/N, T69 insertions, K70E/Q/N/R/T, L74V/I, Y115F, Q151M, M184V/I, L210W, T215F/Y, and K219E/N/Q/R.

**Figure 1:**
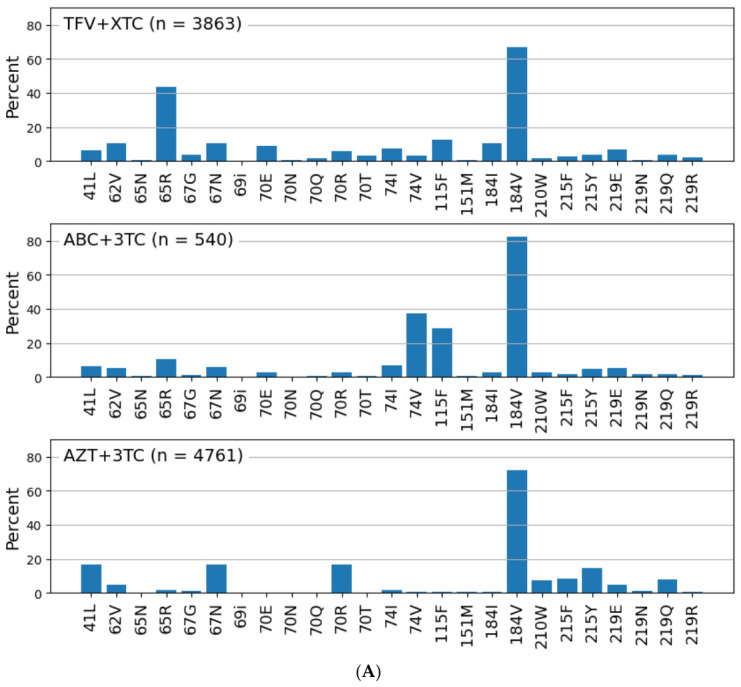

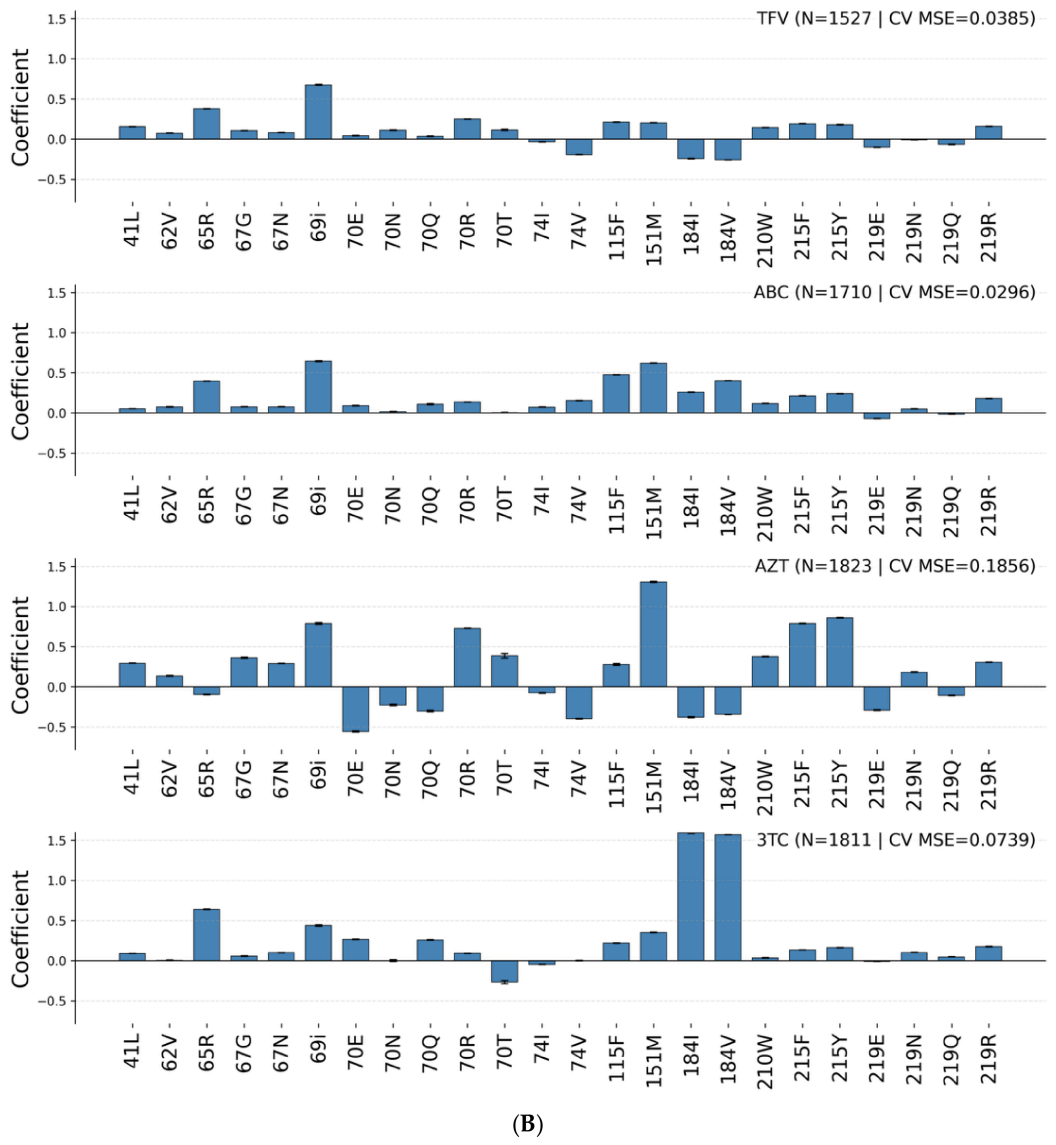
Prevalence (**A**) and In vitro susceptibility (**B**) associated with nucleoside RT inhibitor (NRTI) drug-resistance mutations (DRMs). (**A**) Prevalence of 25 NRTI-associated DRMs at 13 RT positions among individuals who received tenofovir plus 3TC or FTC (TFV/XTC), abacavir plus 3TC (ABC/3TC), and zidovudine plus 3TC (AZT/3TC) and no other NRTI and had at least one NRTI DRM. (**B**) NRTI DRM predictors of reduced TFV, ABC, AZT, and 3TC susceptibility determined by least squares regression in which the DRMs were explanatory variables and the log10-fold reduction in susceptibility was the outcome variable. Sequences containing mixtures at ≥1 DRM positions were excluded from analysis. Ten repetitions of 5-fold cross-validation were performed to estimate the variance among the fitted coefficients. Parentheses contain the number of isolates undergoing PhenoSense testing and the mean-squared error associated with the analysis are shown in the upper right corner of each panel.

Prevalence data: HIVDB contained 6026 HIV-1 isolates from PLWH receiving TFV plus XTC, 809 from those receiving ABC plus 3TC, and 5361 from those receiving AZT plus 3TC. Among these, 64.1% (n = 3863), 66.7% (n = 540), and 88.8% (n = 4761), respectively, had at least one NRTI DRM. Figure 1A shows the prevalence of the 25 NRTI DRMs in PLWH receiving each dual NRTI combination.

M184V/I are the most frequently occurring NRTI DRMs associated with each dual NRTI combination. Among PLWH receiving TFV/XTC, the next most commonly selected DRMs were K65R, Y115F, A62V, and K70E, whereas among those receiving ABC/3TC they were L74V, Y115F, and K65R. The thymidine analog mutations (TAMs) M41L, D67N, K70R, L210W, T215F/Y, and K219E/Q were the most commonly selected DRMs in PLWH receiving AZT/3TC. A low background prevalence of TAMs was also observed among PLWH receiving TFV/XTC and ABC/3TC. It is uncertain whether the TAMs were selected by these regimens or reflect transmitted resistance or incomplete treatment histories. T69 insertions and Q151M were rare, occurring in approximately 0.1% and 0.4% of individuals, respectively.

Drug susceptibility data: PhenoSense (susceptibility test results for TFV (n = 1527), ABC (n = 1710), AZT (1823), and 3TC (1811) were available for analysis. Figure 1B shows the magnitude of the coefficients associated with each DRM by least squares regression. Table 1 summarizes the median fold reductions in susceptibility to TFV, ABC, AZT, and 3TC for viruses with the most common and illustrative NRTI DRM patterns.

M184I/V moderately reduces ABC susceptibility while increasing AZT and TFV susceptibility (Figure 1B, Table 1). K65R reduced TFV and ABC susceptibility but increased AZT susceptibility. L74V and Y115F, which were selected primarily by ABC, reduced ABC susceptibility especially with M184I/V [31,32,33]. Several additional TFV-selected mutations, including A62V, K70E/N/Q/T, L74I, and Y115F were associated with small reductions in TFV susceptibility, either alone or in combination with other common TFV DRMs. K65N is a rare TFV-selected variant with an effect on TFV and ABC susceptibility similar to K65R (Table 1) [34].

Several TAM combinations involving T215F/Y are associated with clinically significant resistance not only to AZT but also to TFV and ABC [32,35]. One of the most common patterns, M41L + L210W + T215Y, conferred intermediate resistance to TFV and ABC, although TFV resistance was partially mitigated by M184I/V [32,35] (Table 1). Other TAMs such as mutations at positions 67 and 70 reduced ABC and TFV susceptibility to a lesser extent than M41L + L210W when present with T215F/Y.

T69 insertions typically occurred in combination with TAMs and conferred high-level resistance to AZT, ABC, and TFV. Q151M usually co-occurred with a characteristic set of mutations (A62V, V75I, F77L, and F116Y) often in combination with K65R and, in this context, conferred high-level resistance to AZT, ABC, and TFV.

### 3.2. INSTI DRMs

We included nonpolymorphic DRMs with a mutation penalty score for DTG, BIC, or CAB and a prevalence ≥ 1% in persons receiving one or more of these INSTIs. Exceptions were made for the rare DRM S153Y because it was selected in vitro by each INSTI. This yielded a set of 23 mutations at 15 positions: A49G, H51Y, T66A/I, E92Q, G118R, E138A/K/T, G140A/R/S, Q146L, S147G, Q148H/K/R, G149A, S153F/Y, N155H, S230R, and R263K.

**Figure 2:**
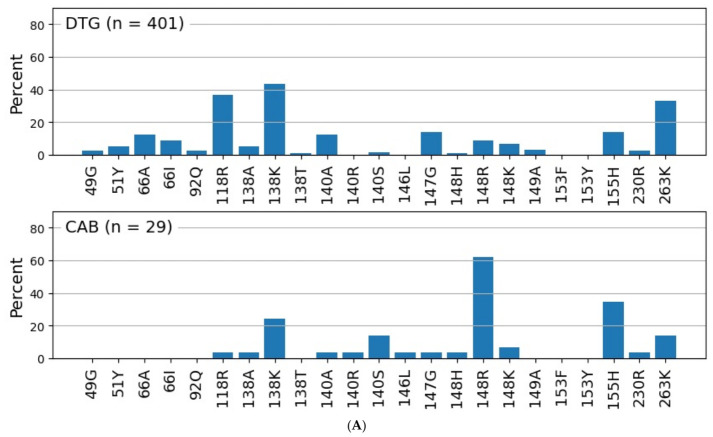

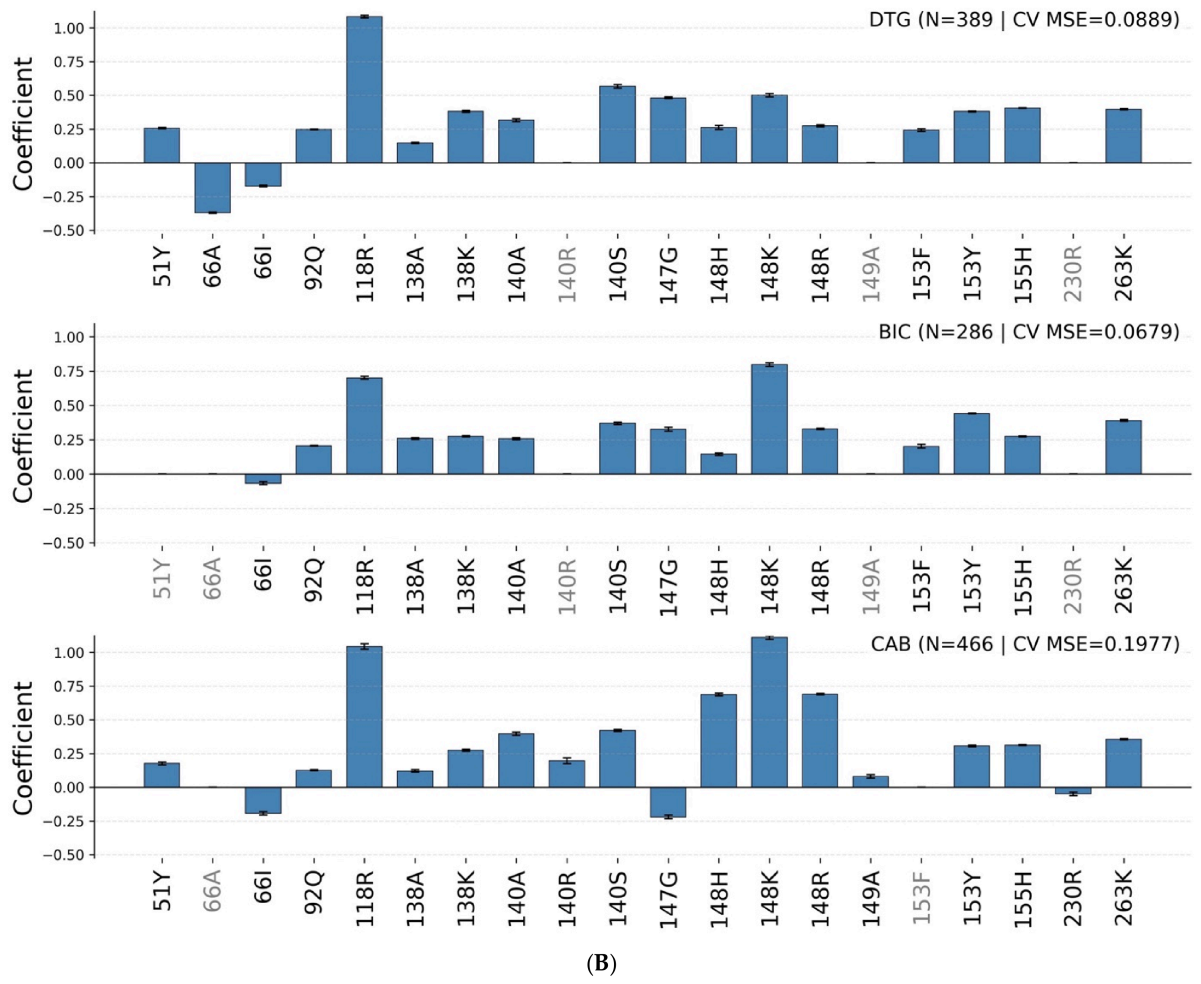
Prevalence (**A**) and in vitro susceptibility (**B**) associated with integrase strand transfer inhibitor (INSTI) drug-resistance mutations (DRMs). (**A**) Prevalence of 23 INSTI-associated DRMs at 15 integrase positions among individuals who received dolutegravir (DTG) or cabotegravir (CAB) and no other INSTI and had ≥1 INSTI DRM. (**B**) INSTI DRM predictors of reduced DTG, bictegravir (BIC), and CAB susceptibility by least squares regression in which the DRMs were explanatory variables and the log_10_-fold reduction in susceptibility was the outcome variable. Sequences containing mixtures at ≥1DRM positions were excluded from analysis. Ten repetitions of 5-fold cross-validation were performed to estimate the variance among the fitted coefficients. Parentheses contain the number of isolates undergoing PhenoSense testing and the mean-squared error associated with the analyses for DTG and BIC are shown in the upper right corner of each panel. For CAB, the number of isolates includes results obtained with PhenoSense and non-PhenoSense assays. Fewer than 3 results were available for the DRMs in a grey font: H51Y, G140R, G149A, and S230R for DTG; H51Y, T66A, G140R, G149A, and S230R for BIC; T66A and S153F for CAB.

Prevalence data: HIVDB contained HIV-1 isolates from 1905 PLWH receiving DTG, 50 receiving CAB, and 7 receiving BIC. Among the complete set of isolates, 21.1% (n = 401) receiving DTG, 58.0% (n = 29) receiving CAB, and 85.7% receiving BIC contained at least one INSTI DRM. Few data on emergent resistance in individuals with VF while receiving BIC have been reported because, unlike DTG, BIC has primarily been used as first-line therapy in combination with one or more fully active NRTIs, usually TFV alafenamide and FTC. Figure 2A shows the prevalence of the 23 INSTI DRMs in PLWH receiving DTG or CAB. Among six PLWH with emergent resistance while receiving BIC, four developed R263K, one developed H51Y, and one developed E138K plus G140S plus Q148H.

G118R, Q148H/K/R, N155H, and R263K are signature DRMs for the second-generation INSTIs because together they occurred in 90.5% (363/401) of viruses associated with VF and emergent INSTI resistance in PLWH receiving DTG and in 96.6% (28/29) of those with VF and emergent INSTI resistance in those receiving CAB [9,10,36,37,38,39,40,41]. Among DTG recipients with any DRM, 37.1% had G118R, 33.8% had R263K, 14.2% had N155H, 8.7% had Q148R, 6.5% had Q148K, and 1.0% had Q148H. Q148H/K/R occurred in combination with E138A/K and/or G140A/S in 95.5% of cases. G118R, N155H and R263K occurred in combination with another DRM in 84.6%, 85.1%, and 34.5% cases, respectively.

Five subtypes accounted for 335 (92.2%) of the 363 sequences with one or more signature DRM: C (60.3%), A (14.3%), B (10.1%), D (8.1%), and CRF02_AG (7.2%). Based on standardized residuals from a chi-square analysis, there was a statistically significant overrepresentation of Q148H/K/R in subtype A, R263K in subtypes B and D, and G118R in subtype C.

Susceptibility data: Susceptibility testing was performed on 968 HIV-1 isolates for DTG, 609 for BIC, and 477 for CAB. Of these, 386 (40.0%) DTG results, 269 (44.2%) BIC results, and 66 (13.8%) CAB results were obtained using the PhenoSense assay. Therefore, PhenoSense data were analyzed for DTG and BIC, whereas all available phenotypic susceptibility data were used for CAB. Figure 2B shows the least squares regression coefficients for each DRM for DTG, BIC, and CAB. Table 2 summarizes the median fold reductions in susceptibility of several of the most common and illustrative INSTI DRM patterns.

G118R caused approximately 8-fold reduced DTG and CAB susceptibility and an estimated 4-fold reduction in BIC susceptibility. R263K was associated with about 2-fold reduced DTG and BIC susceptibility and 3-fold reduced CAB susceptibility (Table 2). Higher reductions in DTG susceptibility were observed in a few isolates when R263K occurred with an additional DRM. Q148R, which frequently emerged in individuals receiving CAB, was associated with a median 5-fold reduction in CAB susceptibility. In contrast, Q148 mutations caused substantial reductions in DTG and BIC susceptibility only when accompanied by additional DRMs. N155H alone had a minimal effect on DTG, BIC, and CAB susceptibility, although high-level resistance occurred in a few viruses harboring multiple additional DRMs.

The nonpolymorphic DRMs E138A/K and G140A/S had little or no impact on DTG, BIC, or CAB susceptibility when present alone. However, when they occurred with Q148 mutations, they increased resistance to each drug (Figure 2B, Table 2). For DTG, the magnitude of this increase ranged from 2-fold to greater than 10-fold. In the VIKING trials, reductions in DTG susceptibility of about 4-fold were associated with reduced virological response to DTG-containing salvage therapy regimens, even when DTG was administered at 50 mg twice daily [42,43].

Among the remaining INSTI DRMs, A49G, H51Y, T66A/I, L74I/M, E92Q, T97A, S147G, G149A, and S230R functioned primarily as accessory mutations that often contributed to incremental reductions in susceptibility when present with a signature INSTI DRM. S153F/Y were selected in vitro by DTG, BIC, and CAB but were rarely observed during INSTI therapy; when present, they conferred approximately 2-fold reduced susceptibility to each drug [44,45,46]. G140R and Q146L each occurred in one person receiving CAB. There are conflicting data on the effect of G140R on CAB susceptibility [47,48].

### 3.3. NNRTI DRMs

We included nonpolymorphic DRMs with an HIVDB mutation penalty score for DOR, ETR, or RPV and a prevalence ≥ 1% among individuals receiving one of these NNRTIs or EFV. Exceptions were made for K103N, which is associated with reduced susceptibility to DOR, ETR, and RPV when present with additional DRMs; for E138A, which is polymorphic but associated with reduced RPV susceptibility; and for L234I which is rare but associated with reduced DOR susceptibility. Five DRMs, which are associated with reduced susceptibility only to EFV (K103S, Y188C/H, and K238T/N) were not included because EFV is rarely considered for salvage therapy in previously treated individuals. This yielded a set of 29 DRMs at 18 positions: A98G, L100I, K101E/P, K103N, V106A/M, V108I, E138A/G/K/Q, V179E/F, Y181C/I/V, Y188L, G190A/E/S, H221Y, P225H, F227C/L, M230L, L234I, P236L, and Y318F.

**Figure 3:**
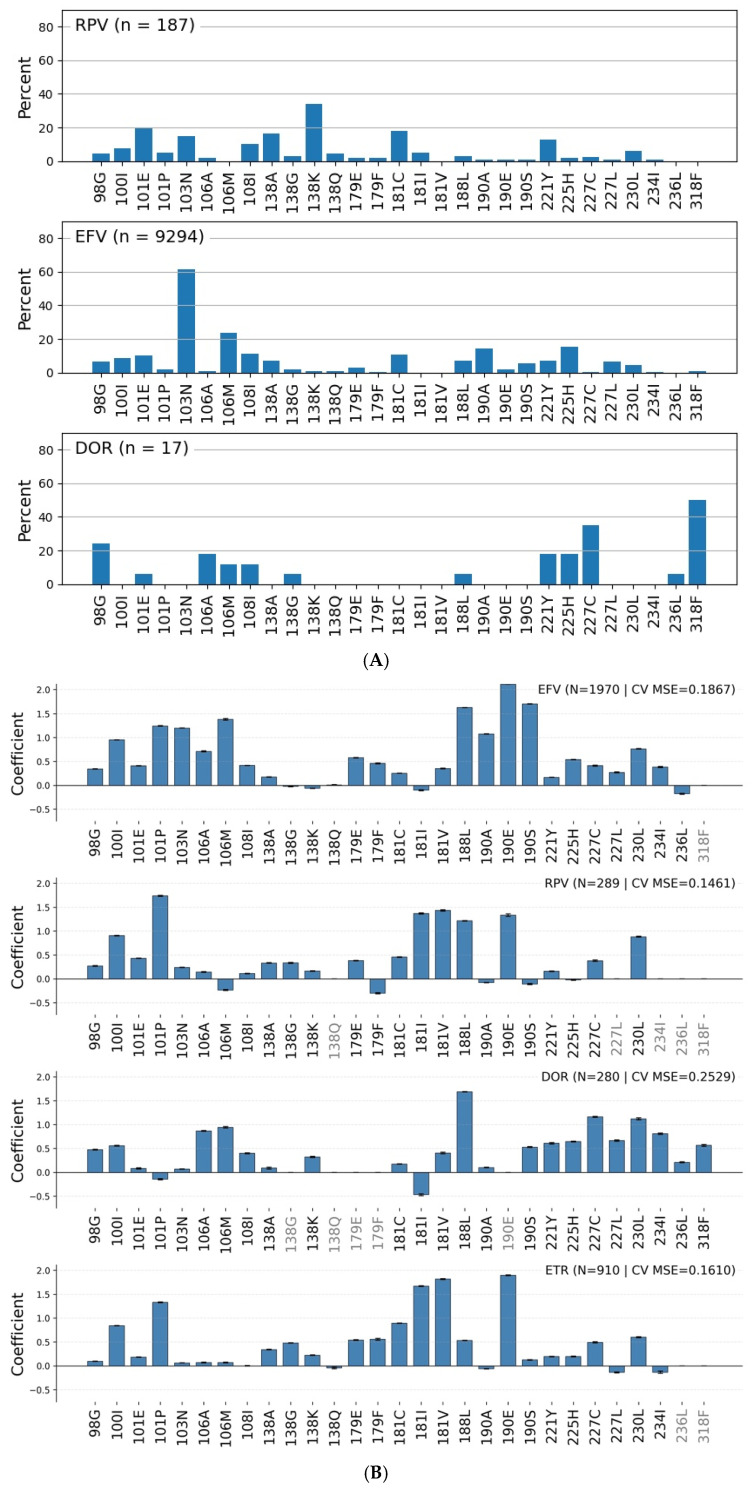
Prevalence (**A**) and in vitro susceptibility (**B**) associated with nonnucleoside RT inhibitor (NNRTI) drug-resistance mutations (DRMs). (**A**) Prevalence of 29 NNRTI-associated DRMs at 18 RT positions among individual who received efavirenz (EFV), rilpivirine (RPV), or doravirine (DOR) and no other NNRTI and had ≥1 NNRTI DRM. (**B**). NNRTI DRM predictors of reduced EFV, etravirine (ETR), RPV, and DOR susceptibility by least squares regression in which DRMs were explanatory variables and log_10_-fold reduction in susceptibility was the outcome variable. Sequences containing mixtures at ≥1 DRM positions were excluded from analysis. Ten repetitions of 5-fold cross-validation were performed to estimate the variance among the fitted coefficients. Parentheses contain the number of isolates undergoing PhenoSense testing and the mean-squared error associated with the analyses for EFV, RPV, and ETR are shown in the upper right corner of each panel. For DOR, the number of isolates includes results obtained with PhenoSense and non-PhenoSense assays. Fewer than 3 results were available for the DRMs in a grey font: Y318F for EFV; E138Q, F227L, L234I, P236L, and Y318F for RPV; E138G/Q, V179E/F, G190E for DOR; P236L and Y318F for ETR.

Prevalence data: HIVDB contained isolates from 12,117 PLWH receiving EFV, 425 receiving RPV, and 17 receiving DOR. The genotypic data for DOR comprised just lists of DRMs as no sequences were available. Because ETR has primarily been used in salvage therapy, few genotypic results were available from individuals receiving ETR as their only NNRTI. At least one NNRTI DRM was detected in 76.7% of isolates from individuals receiving EFV, 44.0% from those receiving RPV, and in all isolates from those receiving DOR. Figure 3A shows the prevalence of the 29 NNRTI DRMs from PLWH receiving either DOR, EFV or RPV. Among 15 PLWH with emergent DRMs while receiving ETR and no other NNRTI, Y181C occurred in 7 individuals, A98G in 3 individuals, and L100I, K101E, and H221Y each in 2 individuals.

Susceptibility data: PhenoSense susceptibility test results for EFV (n = 1970), ETR (n = 910), RPV (n = 289), and DOR (n = 119) were available for analysis. Because relatively few PhenoSense results were available for DOR, the regression analysis for this drug incorporated PhenoSense and non-PhenoSense assay data, yielding a total of 280 susceptibility results. Figure 3B shows the least squares regression coefficients for each DRM for EFV, ETR, RPV, and DOR. Table 3 summarizes the median fold reductions in susceptibility associated with several of the most common and illustrative NNRTI DRM patterns.

Reductions in susceptibility exceeding threefold are uncommon among naturally occurring variants for each of the NNRTIs [29,49,50,51]. For EFV, the DRMs L100I, K101P, K103N, V106A/M, Y188L, and G190A/S/E were each associated with ≥3-fold reductions in susceptibility. For ETR, ≥3-fold reductions were observed with K101P, Y181C/I/V, G190E, K103N + L100I, K103N + M230L, and L100I + Y188L. In the DUET trials, which evaluated ETR-based salvage therapy in individuals with pre-existing NNRTI resistance, estimated lower and upper clinical cutoffs for phenotypic resistance were 3- and 13-fold, respectively [51].

For RPV, ≥3-fold reductions in susceptibility were associated with K101P, Y181I/V, Y188L, G190E, M230L, L100I + K103N, K101E + E138K, and V108I + Y181C. K101E and E138A/G/K/Q each reduced RPV susceptibility by approximately 2-fold and, given RPV’s lower genetic barrier to resistance, are often considered clinically relevant by themselves [52,53]. Although M230I was commonly selected by RPV in vitro and although it has reduced RPV susceptibility by approximately 5-fold in a non-PhenoSense assay [54], it has only been reported in one individual receiving RPV.

Among the limited number of viruses from PLWH experiencing VF on DOR-containing regimens, the DRMs V106A, Y188L, F227C/L, and Y318F were associated with ≥10-fold reductions in DOR susceptibility, either alone or, in the case of F227C/L, in combination with an accessory DRM [49,55]. In addition, M230L and L234I—both selected by DOR in vitro but not observed among the 17 individuals with available data—were associated with ≥10-fold reduced susceptibility [56,57]. Although G190E has also not been reported in PLWH receiving DOR, it similarly confers ≥10-fold reduced susceptibility in vitro [58]. Although, K103N had no measurable effect on DOR susceptibility when present alone, it was associated with about 5-fold reduced DOR susceptibility when combined with either L100I or P225H (Table 3). Insufficient data exist on the phenotypic impact of V106M.

### 3.4. PI DRMs

We included nonpolymorphic DRMs with a mutation penalty score for ATV, DRV, or LPV, and a prevalence ≥ 1% in PLWH receiving one or more of these PIs. An exception was made for the rare mutation I54M because of its association with reduced DRV susceptibility. This yielded a set of 26 DRMs at 19 positions: L10F, K20T, L24I, V32I, L33F, M46I/L, I47A/V, G48V, I50L/V, F53L, I54L/M/V, G73S, T74P, L76V, V82A/F, I84V, N88S, L89T/V, and L90M.

**Figure 4:**
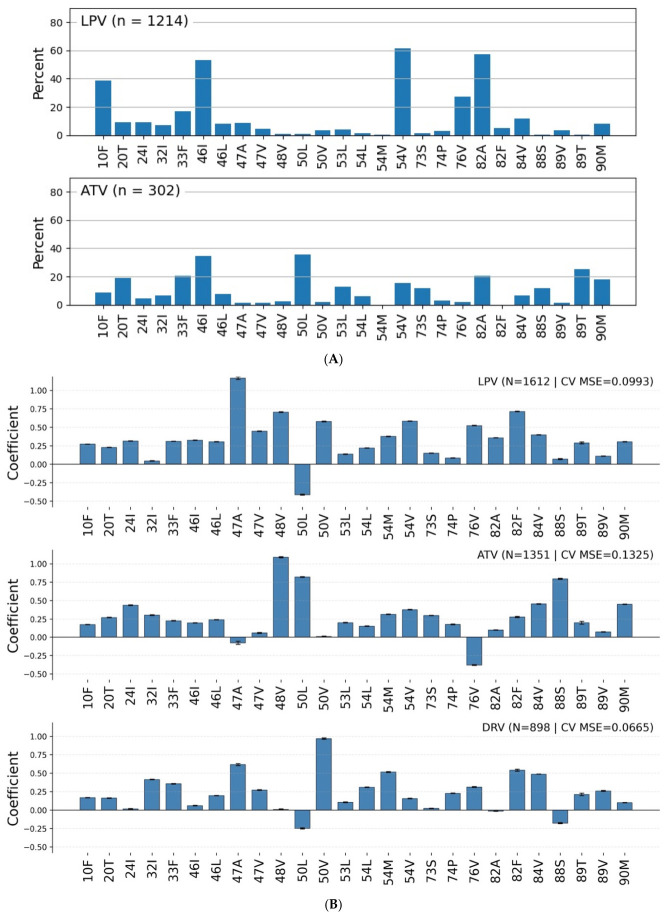
Prevalence (**A**) and in vitro susceptibility (**B**) associated with protease inhibitor (PI) drug-resistance mutations (DRMs). (**A**) Prevalence of 26 PI-associated DRMs at 19 protease positions among individuals who received boosted lopinavir (LPV), atazanavir or boosted atazanavir (ATV), or boosted darunavir (DRV) and no other PI and had ≥1 PI DRM. (**B**) PI DRM predictors of reduced LPV, ATV, or DRV susceptibility by least squares regression in which the DRMs were explanatory variables and the log_10_-fold reduction in susceptibility was the outcome variable. Sequences containing mixtures at ≥1DRM positions were excluded from analysis. Ten repetitions of 5-fold cross-validation were performed to estimate the variance among the fitted coefficients. The parentheses contain the number of isolates undergoing PhenoSense testing and the mean-squared error associated with the analysis are shown in the upper right corner of each panel.

Prevalence data: HIVDB contained 4978 isolates from PLWH receiving LPV, 1183 from those receiving ATV, and 242 from those receiving DRV. At least one PI DRM was present in 24.4% (n = 1214) of isolates from LPV recipients, 25.5% (n = 302) from ATV recipients, and 6.6% (n = 16) from DRV recipients. Figure 4A shows the prevalences of the 26 PI DRMs among PLWH receiving LPV or DRV. DRM prevalence data for DRV are not shown in the figure because only 16 isolates had DRMs.

Susceptibility data: PhenoSense susceptibility test results for LPV (n = 1612), ATV (n = 1351), and DRV (898) were available for analysis. Figure 4B shows the least-squares regression coefficients for each DRM for ATV, DRV, and LPV.

Two partially overlapping resistance pathways to LPV have been described. One is characterized by M46I, I54V, and V82A (Figure 4A), which does not confer cross-resistance to DRV [59,60,61]. Another involves one or more of the DRMs V32I, I47V/A, I50V, L76V, V82F, and I84V which are associated with DRV cross-resistance. Because LPV has a high genetic barrier to resistance, resistance typically emerges after prolonged exposure [62] and is frequently characterized by the accumulation of multiple DRMs.

Although ATV has a lower genetic barrier to resistance compared with LPV, it is better tolerated and as effective as LPV for second-line therapy in LMIC settings [63,64]. ATV selects for two characteristic DRMs I50L and N88S, each of which substantially reduce susceptibility but do not confer cross-resistance to LPV or DRV. However, multiple additional DRMs selected at lower frequencies collectively reduce ATV susceptibility [65].

DRV has become the preferred PI for second-line therapy, and has long been the recommended option for individuals experiencing VF on either LPV or ATV [17,66]. The DRV resistance profile has been defined through in vitro susceptibility testing and analyses of virological response in the POWER trials [67,68]. These studies identified V32I, I47V, I50V, I54L/M, L76V, and I84V as major DRMs that reduce DRV susceptibility and impair treatment response, in conjunction with accessory DRMs including V11I, L33F, G73S, T74P, and L89V. DRV has the highest genetic barrier to resistance among current ARVs, with high-level resistance generally requiring combinations of two to three major DRMs together with two to three accessory DRMs [67,68]. Since the initial description of the DRV DRMs in the POWER trials, three additional DRMs, including L10F, I47A, and V82F, have also been associated with reduced DRV susceptibility [69,70,71].

### 3.5. LEN DRMs

Mutations at seven positions associated with reduced LEN susceptibility have been reported in either in vitro passage experiments or the approximately 25 PLWH receiving LEN including L56I, M66I, Q67H/K/N/Y, K70H/N/R/S, N74D/H/K/S/T, A105S/T, and T107A/C/N [72,73,74]. Among these DRMs, M66I, Q67H, and N74D are the most common and can occur alone or with DRMs at other positions. Among 40 isolates from the 25 PLWH in HIVDB for which mutation data are available, M66I was present in 15 isolates, Q67H in 13 isolates, and N74D in 9 isolates. The remaining DRMs occurred in combination with one or more DRMs at these three positions. L56I has only been selected in vitro.

Individually, Q67H, N74D, and M66I were associated with median 6-fold, 17-fold, and >1000-fold reductions in susceptibility [73,74,75]. In combination with each other, Q67H and N74D were also associated with >1000-fold reduced susceptibility. In combination with accessory DRMs, Q67H was associated with 18 to 66-fold reduced susceptibility. K70H and K70N have been reported to reduce LEN susceptibility by 154-fold and 24-fold in site-directed mutants. Alone K70R does not reduce LEN susceptibility but in combination with Q67H it leads to nearly 20-fold reduced susceptibility.

### 3.6. WHO TPP Scenario 1: Confirmed VF in PLWH Receiving an INSTI-Containing Regimen (Table 4)

In this scenario, the primary goal of genotypic resistance testing is to distinguish VF due to suboptimal adherence from VF due to drug resistance. Many PLWH in this setting will have had prior exposure to NRTI-containing regimens, most commonly a WHO-recommended first-line NRTI/NNRTI regimen. Because TLD has demonstrated high efficacy even in the presence of common NRTI DRMs, including M184I/V with or without K65R, the detection of NRTI resistance alone is insufficient to attribute VF to drug resistance [76]. Given that more than 90% of individuals with VF and emergent INSTI resistance harbor one or more signature DRMs at positions 118, 148, 155, or 263, a genotypic assay capable of detecting mutations at these positions would be highly informative in this context [9,10,37].

Another challenging question is whether PLWH with evidence of emergent INSTI resistance should be switched to a PI-containing regimen. This has important programmatic implications, as the recommended PI, typically DRV, is substantially more expensive than DTG [77]. With the exception of G118R, the presence of a single signature INSTI DRM is generally associated with ≤2-fold reduced DTG susceptibility. However, Q148 mutations and N155H nearly always occurred in combination with other nonpolymorphic DRMs. R263K is notable because it usually occurs in isolation. Whether continued DTG-based therapy remains effective in the presence of certain DTG-resistance mutations is the subject of ongoing investigations, such as in the Ndovu Study [78].

### 3.7. WHO TPP Scenario 2: Confirmed VF in Heavily Treated PLWH Who Have Received PIs (Table 4)

For individuals with VF following treatment with a first-line NRTI/NNRTI regimen and subsequent PI-based therapy, genotypic testing of RT and protease will be sufficient, as transmitted INSTI resistance remains exceedingly uncommon. However, among individuals with a history of VF following treatment with NRTIs, NNRTIs, PIs, and INSTIs, genotypic testing of RT, protease, and integrase will generally be required.

Priority NRTI DRMs: The detection of M184I/V and/or K65R can help determine the degree of residual activity for TFV and the number of additional ARVs required to achieve sustained virological suppression. In infants and young children, the detection of K65R, L74V, and/or Y115F in combination with M184V/I indicates high-level ABC resistance and that NRTIs may not contribute substantially to salvage therapy [31,32,33]. T215Y/F may be present in PLWH who received a thymidine analog. Whereas T215Y/F alone is not sufficient to substantially reduce TFV and ABC susceptibility, its detection by a point mutation assay would indicate the possible presence of a TAM pattern associated with intermediate levels of TFV and ABC resistance.

Priority INSTI DRMs: Because DTG resistance most commonly arises through a limited set of INSTI DRMs, the same mutations highlighted for TPP scenario 1, G118R, Q148H/K/R, N155H, and R263K, are also likely to be the most informative for deciding whether to include DTG or BIC in a salvage therapy regimen for heavily treated individuals. As previously noted, G118R, Q148H/K/R and, to a lesser extent, N155H usually present in combinations expected to result in clinically significant reductions in DTG and BIC susceptibility while the clinical significance of R263K for salvage therapy with DTG or BIC has not been studied.

Priority NNRTI DRMs: In some LMICs, the second-generation NNRTI ETR has been used for third-line therapy in PLWH previously treated with a first-generation NNRTI followed by a PI-containing regimen [13,14,15,18,19,20]. Although RPV has a similar resistance profile, it has a lower genetic barrier to resistance than ETR and is less widely available in most LMICs. DOR is also not yet widely available in LMICs but may become more accessible in the future.

The most common DRM associated with ETR resistance was Y181C, which conferred approximately 4-fold reduced susceptibility. Several less common DRMs, including K101P, Y181I/V, G190E, and M230L, as well as certain DRM combinations involving L100I or Y188L, were also associated with clinically significant reductions in ETR susceptibility.

The DRMs most clearly associated with reduced DOR susceptibility included Y188L and the less common mutations V106A, G190E, F227C/L, M230L, and Y318F. Additional DRMs prevalent in those receiving a first-generation NNRTI, including L100I, V106M, and P225H also reduce susceptibility especially in combination with other DRMs, although further data are needed to better define their effects.

Taken together, these data indicate that many of the NNRTI DRMs most relevant to selecting a salvage regimen with either ETR (L100I, K101P, Y181C/I/V, Y188L, G190E, and M230L) or DOR (L100I, V106A/M, Y188L, G190E, F227C/L, M230L, and Y318F) are uncommon. Further complicating assay design, combinations of three or more NNRTI DRMs can produce reductions in susceptibility that are difficult to predict from individual mutations alone [79].

Priority PI DRMs: Most PI-experienced PLWH are expected to have been treated with LPV or ATV, with DRV being the principal PI option for salvage therapy. As outlined above, a substantial proportion of individuals receiving LPV will exhibit partial cross-resistance to DRV resulting from V32I, I47A/V, I50V, I54L/M, L76V, V82F, and/or I84V [59,60,61]. DRV will usually retain considerable activity in treating individuals who received ATV. Assays designed to detect individual PI DRMs should prioritize the major DRV DRMs.

### 3.8. Additional WHO TPP Scenario: Individuals Who Test Positive for HIV While Receiving PrEP (Table 4)

Currently available PrEP regimens include TFV/XTC and the long-acting agents CAB and LEN. Among individuals who acquire HIV infection while receiving TFV/XTC for PrEP, the most commonly reported DRMs have been M184I/V, detected in approximately 20% of cases, and K65R, detected in approximately 1% [80]. Among individuals who acquired infection while receiving CAB in the HPTN 083 trial and had an INSTI DRM, all developed one of the four signature INSTI DRMs usually in combination with an additional DRM [81,82]. Four virological breakthrough infections with resistance have been reported during LEN PrEP, including three associated with the capsid mutation N74D and one associated with Q67H [83,84].

Based on these data, the most informative DRMs for this scenario include M184I/V and K65R for individuals receiving TFV/XTC and G118R, Q148H/K/R, N155H, and R263K for those receiving CAB. Additional data, likely to emerge from infrequent case reports of individuals mistakenly receiving LEN during acute HIV-1 infection, will be required to more fully define the resistance profile associated with LEN PrEP failure. In the interim, it may be prudent for developers of point-mutation assays to also detect M66I which is commonly reported among individuals with VF while receiving LEN and which is associated with high-level LEN resistance in vitro [73].

## 4. Conclusions

This review provides an evidence-based framework for prioritizing testing HIV-1 DRMs across five major ARV drug classes currently recommended by the WHO for the treatment or prevention of HIV. By integrating prevalence and phenotypic susceptibility data, the analysis has identified a limited set of high-value DRMs that are most likely to inform the design of cost-effective genotypic resistance assays suitable for LMICs. These data complement the WHO TPP for HIVDR testing and are intended to support diagnostic manufacturers focus on DRMs with the greatest clinical relevance.

For NRTIs, INSTIs, and LEN, resistance is largely driven by a small number of recurrent mutations, suggesting that targeted or multiplexed assays could capture most clinically relevant resistance. In contrast, NNRTI and PI resistance are more genetically diverse, implying that broader sequencing approaches may be required. However, if a particular NNRTI was being considered for therapy, an adaptive approach focusing on a smaller set of DRMs would still provide clinically actionable guidance.

The analyses presented here were often constrained by the limited availability of publicly accessible sequence and phenotypic data, particularly for newer agents such as CAB, BIC, and DOR. Finally, clinical studies linking individual DRMs or DRM patterns to virological response remain limited. This distinction is important because reductions in in vitro susceptibility do not necessarily translate into proportional reductions in antiviral activity in vivo, where drug exposure, potency, and pharmacologic properties also contribute to treatment efficacy. Addressing these gaps through increased public availability of sequence, phenotypic, and clinical outcome data will be essential for refining and validating the DRMs highlighted in this review.

## Figures and Tables

**Table 1:** Median Fold Reductions in NRTI Susceptibility Associated with Selected NRTI DRM Patterns.

DRM Pattern	AZT (Median-Fold Reduction)	3TC (Median-Fold Reduction)	ABC (Median-Fold Reduction)	TFV (Median-Fold Reduction)
***Discriminatory mutations***				
M184V	0.5_(123)_	>200_(139)_	3.1_(122)_	0.5_(67)_
K65R	0.5_(17)_	9.1_(20)_	2.4_(16)_	1.8_(16)_
K65N	-	7.3_(1)_	2.1_(1)_	1.7_(1)_
L74V	0.4_(5)_	1.9_(7)_	2.1_(3)_	0.6_(4)_
Y115F	0.9_(1)_	1.6_(1)_	2.8	1.3_1_
K70E	-	3.4_(1)_	1.4_(1)_	1.4_(1)_
A62V, K65R	0.5_(1)_	6.3_(1)_	1.6_(1)_	1.8_(1)_
K65R, Y115F	0.3_(2)_	22_(1)_	5.8_(2)_	1.8_(2)_
K65R, M184V	0.3_(6)_	>200_(6)_	8.5_(6)_	1.0_(6)_
L74V, M184V	0.3_(8)_	110_(9)_	5.7_(7)_	0.6_(4)_
K70E, M184V	0.2_(4)_	>200_(4)_	3.4_(4)_	0.6_(4)_
Y115F, M184V	0.7_(3)_	>200_(3)_	11_(3)_	0.9_(3)_
***Thymidine analog mutations (TAMs)***				
T215Y	8.1_(15)_	1.9_(1)_	1.6_(11)_	1.3_(14)_
T215F	2.9_(3)_	1.1_(3)_	1.0_(3)_	0.9_(3)_
K70R	3.1_(11)_	1.2_(3)_	1.0_(12)_	1.1_(8)_
M41L, T215Y	12_(10)_	1.8_(12)_	2.3_(9)_	1.3_(7)_
M41L, L210W, T215Y	138_(14)_	2.5_(17)_	3.1_(13)_	3.1_(12)_
M41L, D67N, L210W, T215Y	>200_(9)_	4.5_(10)_	5.3_(9)_	5.1_(7)_
D67N, K70R, K219Q	15_(15)_	1.8_(18)_	1.8_(13)_	2.1_(13)_
D67N, K70R, T215F, K219Q	171_(12)_	3.2_(12)_	2.8_(10)_	2.6_(12)_
***TAMs plus M184V***				
T215Y, M184V	1.3_(13)_	>200_(14)_	5.2_(13)_	0.4_(6)_
M41L, T215Y, M184V	6.0_(31)_	>200_(32)_	5.2_(32)_	1.1_(19)_
M41L, L210W, T215Y, M184V	14_(37)_	>200_(38)_	6.2_(34)_	1.4_(32)_
D67N, K70R, K219Q, M184V	3.0_(22)_	>200_(29)_	4.1_(23)_	1.2_(19)_
***Multidrug-resistance mutations***				
Q151M	2.9_(3)_	1.4_(3)_	3.7_(3)_	0.9_(2)_
V75I, F77L, F116Y, Q151M	72_(2)_	2.0_(2)_	7.0_(2)_	1.8_(2)_
A62V, K65R, V75I, F77L, F116Y, Q151M	>200_(1)_	36_(1)_	25_(1)_	8_(1)_
T69_insertion_, M41L, L210W, T215Y	>200_(3)_	25_(3)_	25_(2)_	12_(3)_

Footnote: Values represent median fold reductions in susceptibility relative to wild-type control viruses. Subscripts indicate the number of isolates tested for each DRM pattern.

**Table 2:** Median Fold Reductions in INSTI Susceptibility Associated with Selected INSTI DRM Patterns.

DRM Pattern	CAB (Median-Fold Reduction)	BIC (Median-Fold Reduction)	DTG (Median-Fold Reduction)
***G118R***			
G118R	8.0_(3)_ *	4.2_(4)_	8.2_(5)_
G118R, H51Y	-	5.0_(1)_	21_(2)_
G118R, E138K	-	-	8.0_(1)_
***R263K***			
R263K	2.5_(10)_ *	1.5_(4)_	2.0_(10)_
H51Y, R263K	4.1_(1)_ *	2.0_(2)_ *	7.0_(1)_
A49G, R263K	-	-	4.2_(1)_
***Q148H/R/K***			
Q148H	1.8_(2)_ *	0.7_(1)_	0.5_(5)_
Q148K	3.1_(3)_ *	0.9_(2)_	0.8_(5)_
Q148R	7.3_(6)_	1.0_(9)_	0.9_(11)_
E138K	1.2_(5)_ *	0.8_(2)_	0.8_(5)_
G140S	1.0_(3)_ *	0.8_(1)_	0.8_(2)_
G140S, Q148H	8.7_(15)_	2.5_(34)_	4.1_(23)_
G140A, Q148R	6.2_(3)_	2.1_(6)_	2.8_(3)_
G140S, Q148R	11_(6)_	3.2_(9)_	5.1_(4)_
E138A, Q148R	12_(2)_ *	1.7_(1)_	2.2_(1)_
E138K, Q148R	8.1_(3)_ *	1.9_(5)_	2.0_(3)_
E138K, Q148K	105_(2)_ *	4.4_(1)_	5.1_(2)_
E138A, G140S, Q148H	59_(2)_	6.0_(4)_	8.7_(2)_
E138K, G140S, Q148H	22_(8)_	2.6_(11)_	5.3_(3)_
E138K, G140A, Q148R	54_(2)_ *	18_(1)_	13_(1)_
E138K, G140A, Q148K	84_(3)_	23_(4)_	63_(1)_
***N155H***			
N155H	1.8_(9)_	1.3_(22)_	1.5_(23)_
E92Q, N155H	2.2_(9)_ *	1.8_(2)_	3.0_(4)_

Footnote: Values represent median fold reductions in susceptibility relative to wild-type control viruses. Subscripts indicate the number of isolates tested for each DRM pattern. Asterisks (*) denote susceptibility results generated using non-PhenoSense assays.

**Table 3:** Median Fold Reductions in NNRTI Susceptibility Associated with Selected NNRTI DRM Patterns.

DRM Pattern	EFV (Median-Fold Reduction)	RPV (Median-Fold Reduction)	ETR (Median-Fold Reduction)	DOR (Median-Fold Reduction)
***Single DRM***				
L100I	9.7_(6)_	0.7_(5)_	1.4_(7)_	1.5_(3)_
K101E	2.5_(14)_	2.1_(9)_	1.3_(9)_	4.5_(1)_
K101P	20_(2)_	23_(2)_	4.8_(5)_	1.5_(3)_ *
K103N	13_(194)_	1_(12)_	0.6_(82)_	1.2_(6)_
V106A	2.1_(4)_	1.2_(2)_	1.1_(2)_	18_(2)_
V106M	55_(2)_	0.5_(1)_	0.7_(2)_	3.4_(1)_
E138A	1.1_(22)_	2.1_(8)_	1.8_(14)_	2.1_(1)_
E138K	1.0_(18)_	1.6_(17)_	1.6_(18)_	0.7_(5)_
Y181C	1.3_(60)_	2.1_(10)_	4.2_(20)_	1.4_(4)_
Y181I	0.6_(6)_	12_(4)_	18_(3)_	1.1_(2)_ *
Y181V	1.6_(6)_	20_(3)_	53_(3)_	5.1_(2)_
Y188L	59_(46)_	6.4_(10)_	1.4_(24)_	149_(6)_
G190A	5.2_(26)_	0.8_(2)_	0.8_(9)_	2.7_(2)_
G190E	>200_(8)_	7.3_(1)_	70_(2)_	18_(1)_
G190S	56_(11)_	0.4_(4)_	0.5_(8)_	5.2_(4)_
M230I	5.4_(4)_ *	5.6_(3)_ *	3.5_(5)_ *	-
Y318F	1.1_(5)_ *	-	1.4_(1)_ *	11_(3)_
***DRM combinations***				
L100I, K103N	>200_(68)_	12_(12)_	4.5_(42)_	6.8_(8)_
L100I, Y188L	>200_(2)_	>200_(1)_	64_(3)_	-
K101P, K103N	>200_(14)_	>200_(5)_	38_(9)_	0.6_(2)_ *
K101E, E138K	2.0_(2)_	3.2_(2)_	3.1_(2)_	0.3_(1)_ *
K103N, Y181C	23_(48)_	2.8_(7)_	6.8_(18)_	4.4_(4)_
K103N, P225H	101_(17)_	1.0_(2)_	0.8_(6)_	7.8_(2)_
K103N, M230L	>200_(7)_	4.0_(2)_	3.5_(2)_	36_(1)_
V106A, P225H	4.8_(1)_	1.0_(1)_	0.7_(1)_	153_(2)_
V106A, F227L	5.8_(8)_	-	0.8_(1)_	106_(1)_
V106I, F227C	2.5_(1)_	3.4_(1)_	4.0_(1)_	105_(1)_
V106A, L234I	-	0.4_(1)_ *	-	161_(2)_ *

Footnote: Values represent median fold reductions in susceptibility relative to wild-type control viruses. Subscripts indicate the number of isolates tested for each DRM pattern. Asterisks (*) denote susceptibility results generated using non-PhenoSense assays.

**Table 4:** High Impact DRMs Across WHO-Defined Scenarios.

WHO TPP Scenario	ARVs	DRMs	Rationale
#1 Confirmed VF in PLWH receiving an INSTI-based regimen (e.g., TLD)	NRTIs	K65R, M184I/V	M184I/V and, to a lesser extent, K65R are likely to be present in most individuals at the time of VF while receiving TLD. However precise prevalence data are not available because most studies have reported only the INSTI DRMs.
	INSTIs	G118R, Q148H/K/R, N155H, R263K	About 90% of PLWH with VF on DTG and a DRM will have ≥1 second-generation INSTI signature DRM. G118R alone reduces DTG susceptibility about 8-fold. Q148H/K/R and N155H minimally reduce susceptibility. Q148H/K/R nearly always occur with additional DRMs at positions 138 and 140. N155H usually occurs with additional DRMs. R263K reduces DTG susceptibility about 2-fold. It usually occurs without other DRMs.
#2 Confirmed VF in heavily treated PI-experienced PLWH	NRTIs	K65R, M184I/V ± L74V, Y115F (history of ABC) ± T215Y (history of AZT or d4T)	Determines the extent of residual activity of associated with the dual NRTI backbone.
	PIs	DRV: V32I, I47A/V, I50V, I54L/M, L76V, V82F, I84V	Pharmacokinetically boosted DRV is the recommended PI for salvage therapy in heavily treatment-experienced PLWH.
	NNRTIs	ETR: L100I, K101P, Y181C/I/V, Y188L, G190E, M230L DOR: L100I, V106A/M, Y188L, G190E/S, P225H, F227C/L, M230L, Y318F	ETR and DOR are likely to retain activity in PLWH with previous VF after receiving a first-generation NNRTI. Each of the DRMs listed with the exception of F227C has been reported in ≥1% of PLWH receiving a first-generation NNRTI. F227C, however, is included because it is one of the most common DRMs arising in PLWH receiving DOR.
	INSTIs	G118R, Q148H/K/R, N155H, R263K	PLWH with G118R, Q148H/K/R, and N155H are likely to harbor additional INSTI DRMs and viruses with these DRMs are likely to have clinically significant reductions in DTG and BIC activity.
#3 Individuals who test positive for HIV while receiving PrEP	TFV/XTC	K65R, M184I/V	K65R has been rare in clinical trials of PrEP but may be more common in routine clinical settings
	CAB	G118R, Q148H/K/R, N155H, R263K	PLWH with G118R, Q148H/K/R, and N155H are likely to harbor additional INSTI DRMs and viruses with these DRMs are likely to harbor clinically significant reductions in DTG and BIC activity.
	LEN	M66I, Q67H, N74D	Q67H and N74D have been reported with PrEP breakthrough. M66I has occurred commonly in persons receiving LEN for therapy.

## Data Availability

All of the data presented in the manuscript including Figure 1, Figure 2, Figure 3 and Figure 4 and Table 1, Table 2 and Table 3 are available in tables in the Stanford HIV Drug Resistance Database (https://hivdb.stanford.edu).
